# Effect of cryotherapy on pain scores and satisfaction levels of patients in cataract surgery under topical anesthesia: a prospective randomized double-blind trial

**DOI:** 10.1186/s13104-022-06125-w

**Published:** 2022-06-28

**Authors:** Marzieh Beigom Khezri, Abbas Akrami, Matina Majdi, Bijan Gahandideh, Navid mohammadi

**Affiliations:** 1 Department of Anesthesiology, Professor of Anesthesiology, Metabolic Diseases Research Center, Research Institute for Prevention of Non-Communicable Diseases, Qazvin University of Medical Sciences, Qazvin, Iran; 2 Department of Ophthalmology, Qazvin University of Medical Sciences, Qazvin, Iran; 3 Department of Anesthesiology, Resident of Anesthesiology, Qazvin University of Medical Sciences, Qazvin, Iran; 4 Department of Nursing, Qazvin University of Medical Sciences, Qazvin, Iran; 5 Children Growth Research Center, Research Institute for Prevention of Non-Communicable Diseases, Qazvin University of Medical Sciences, Qazvin, Iran; 6 Researcher, Canada Optimax Access Consulting, Port Coquitlam, BC, Canada

**Keywords:** Cryotherapy, Phacoemulsification, Topical anesthesia, Cataract

## Abstract

**Objective:**

To evaluate the effects of cryotherapy on pain scores and satisfaction levels of patients during cataract surgery under topical anesthesia. Eighty patients aged between 55 and 75 years scheduled for cataract surgery were randomly allocated to two study groups to receive topical anesthesia with cryotherapy (TC) or topical anesthesia alone (T) groups. Visual analog pain scores, patient satisfaction level, hemodynamic parameters, and quality of operating conditions were recorded.

**Results:**

Cryotherapy significantly reduced VAS pain scores during surgery (P = 0.014). Although no significant difference in postoperative pain scores, opioid consumption, heart rate, and mean arterial blood pressure was seen in the postoperative period. The surgeon reported better quality of operating conditions in the TC group (*P* = 0.018). Cryotherapy as a complementary method with topical anesthesia reduced pain scores of patients during surgery. It also produced a better quality of operating conditions for surgeons. There was no significant difference in either postoperative pain scores or opioid consumption.

*Trial registration* This trial was registered at Iranian clinical trial registering: IRCT registration number: IRCT2017052734091N2

**Supplementary Information:**

The online version contains supplementary material available at 10.1186/s13104-022-06125-w.

## Introduction

Cataract surgery is one of the most common surgical procedures in men and women aged between 50 and 80 [1]. The most common method for cataract surgery is phacoemulsification with topical anesthesia [2]. Topical anesthesia protects patients from the possibility of globe perforations, optic nerve injury, and risk of respiratory arrest [2, 3]. Nowadays, systemic analgesic and hypnotic agents are used to relieve patient distress and enhance the patient's satisfaction with surgery [3]. However, these agents may cause disorientation, respiratory depression, hypotension, bradycardia, and cardiovascular depression. Furthermore, each of these agents may lead to impairment of the patient’s cooperation during surgery and work less than ideal agents for the management of conscious sedation [1–3]. Therefore, the potential clinical benefits of the new approach in this setting need to be assessed.

The analgesic role of low temperatures during cataract surgery, even without the association of topical anesthetics was described [4, 5]. The possible action mechanism of cryoanalgesia in reducing pain is related to a decrease in the activities in the polymodal sensitive neurons [6, 7]. Thus, during phacoemulsification with cold intraocular irrigation solution, the cold would reduce the transmission of painful mechanical stimulus [4, 5]. On the other hand, another study reported a dubious benefit of cryoanalgesia in phacoemulsification, but no difference in the severity of pain during phacoemulsification with topical anesthesia either with or without cryoanalgesia [8].

Due to the controversial reports about the analgesic effects of cryotherapy during cataract surgery, the present study was conducted to compare patient-reported pain intensity during cataract surgery with topical anesthesia versus topical combined to cryotherapy.

## Main text

### Materials and methods

In a prospective, double-blinded randomized trial, 80 patients were recruited for cataract surgery by phacoemulsification after the approval of the Institutional Ethics Committee and written informed consent. The consolidated standards of reporting trials (CONSORT) recommendations for reporting randomized controlled clinical trials were followed. Inclusion criteria were patients aged 55–75 years scheduled for elective cataract surgery with intraocular lens implantation using phacoemulsification under topical anesthesia for the first time according to the American Society of Anesthesiologists (ASA) physical status classification I–III. Exclusion criteria were patients with sleep disorders, depressive disorder, or expected compliance problems (known psychiatric disease), epilepsy, insufficient pupillary dilation, nystagmus, deafness, ongoing treatment with hypnotics or psychotropic drugs (including opioids) within a week before admission, daily analgesic NSAIDs treatment. All patients received 3 mg of melatonin one hour before entering the operating room to reduce anxiety.

Eighty patients aged between 55 and 75 scheduled for cataract surgery were randomly allocated to two study groups to receive either topical anesthesia with cryotherapy (TC) or topical anesthesia alone (T) using a computer-generated randomization schedule. Allocation was managed by a resident external to the project and the study drugs given by a nurse noninvolved in the study. The anesthetist was blinded to the patient’s group assignment, and the study data were recorded by a blinded observer. All solutions to be instilled during the operation, except the povidone drops, were cooled to around 4C. Mydriasis was achieved by tropicamide 1%. No patient received preoperative sedation. An eye mask of cold gel (Eyes Pack Single) was placed over the eye for about 10 min before surgery. After washing the periocular skin with chlorhexidine 0.5%, ocular asepsis was achieved using povidone 5% drops. The intraocular irrigation was performed by a solution at room temperature (approximately 23 °C) in group T and 4 °C in group TC. The solution for group TC was kept in a refrigerator at approximately 4 °C temperature. The storage advised by the manufacturer was 2–27 °C. The same surgeon performed all surgeries. The lens opacity was almost the same in both groups, according to the LOCS.

Phacoemulsification was performed through a temporal clear corneal incision technique. An applicator was used to hold the globe to avoid touching the conjunctiva or sclera. In group TC, during phacoemulsification, the cornea was kept chilled with cold BSS. The lens nucleus was hydro-dissected with cold BSS irrigation.

No other sedative or analgesic agent was used. At the preoperative visit, the Visual analogue pain scores (VAS) ranged from 0 to 10 (0 = no pain and 10 = worst pain imaginable). Patients were monitored by an electrocardiogram, noninvasive measurement of blood pressure, and pulse oximetry (SPO2). Pain scores were recorded for each patient several times: before premedication (T1), 60 min after premedication, on arrival at the operating room (T2), during the operation period (T3), and also postoperatively before discharge from the recovery room (T4). The primary outcomes of this prospective, double-blinded randomized trial were to evaluate the severity of patient’s pain and satisfaction level during the operation period. At the end of the surgery, the patients were asked about the severity of their pain during the operation period according to the VAS criteria explained before premedication.

After surgery, the sedation level of patients was measured according to the modified Ramsay score scale using a 3-point scale with 1 = anxious, 2 = calm and oriented, 3 = calm and drowsiness.

Complications such as respiratory depression, headache, nausea, vomiting, and chills were also recorded.

Postoperatively, the operating surgeon, who was unaware of patient assignment, was asked to assess the adequacy of intraoperative conditions according to the following scale: excellent (complete calmness and cooperating with the surgeon), good (slight undesirable movements of the eye, and poor (severe undesirable movements of the eye and un-cooperating). Patient satisfaction was also assessed by the Iowa Satisfaction with Anesthesia Scale (ISAS) [9, 10]. The secondary outcome of this study included the assessment of complications such as respiratory depression, headache, nausea, vomiting, and chills.

According to a previously published study [8], considering a significance level of 5% and power of 80%, 50 patients should be included (25 per group) to the minimum difference between groups become significant. Parametric data were expressed as the mean ± SD. The normality of the distribution was tested by a one-sample Kolmogorov–Smirnov test. The *t*-test analysis was used for continuous parametric variables. Nonparametric data were expressed as the median interquartile range. The pain scores and patient satisfaction were compared between groups by Fisher’s exact test; otherwise, the Mann–Whitney U test was used. A *P* value < 0.05 was considered significant. Statistical analysis was carried out using SPSS version 16 for Windows (SPSS, Chicago, IL).

### Results

**Fig. 1:**
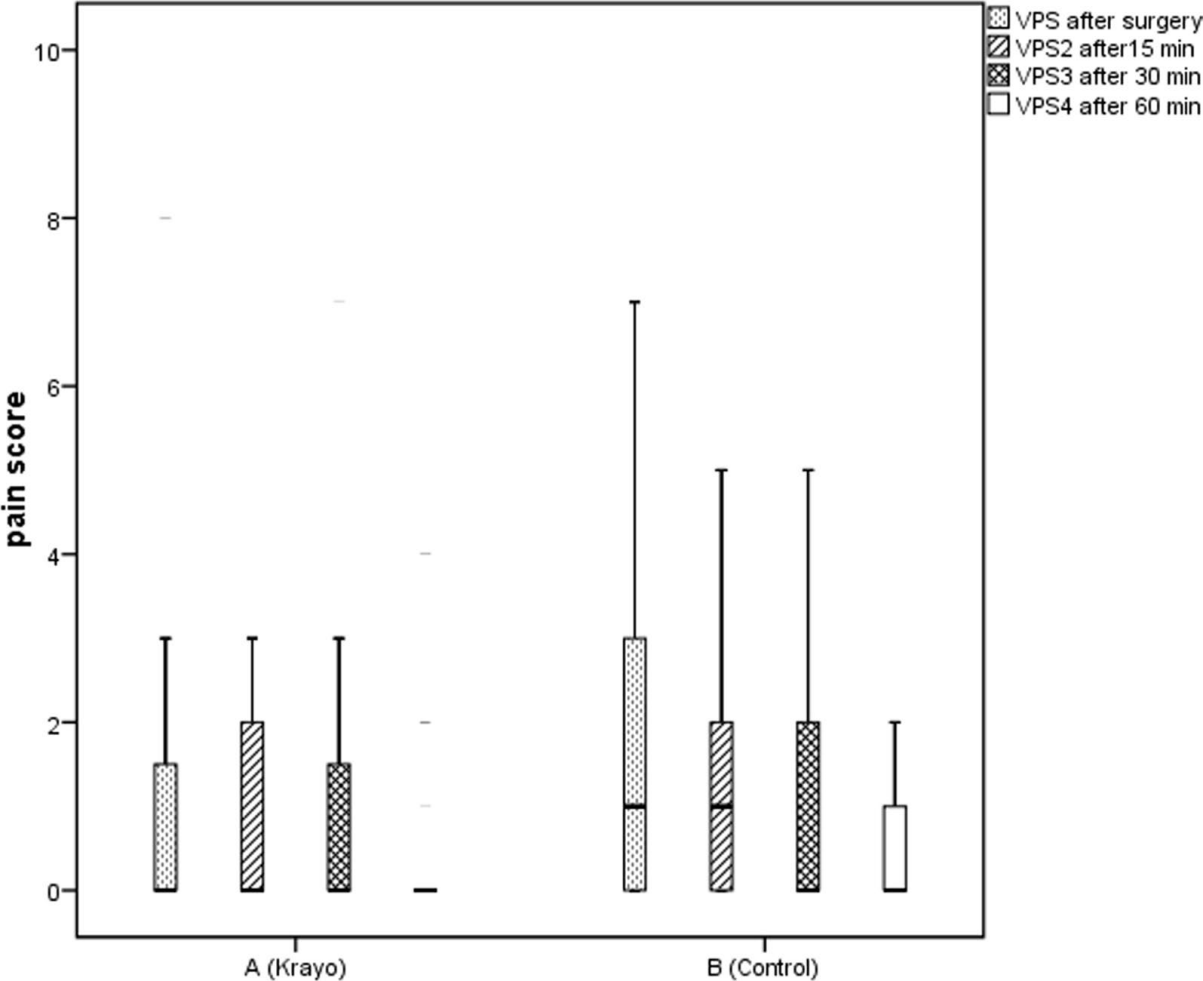
Intensity of pain in the two groups in 15, 30, 60 min after surgery

We excluded ten out of 90 patients due to logistical issues or other violations of the study protocol. Eighty patients were randomly assigned to two groups. There were no significant differences between the two groups regarding the demographic features including age, gender, and duration of surgery [Data are in Additional file 1: Fig. S1, Additional file 2: Table S1, Additional file 3: Table S2, Additional file 4: Table S3, Additional file 5: Table S4]. Figure 1 shows, the intensity of pain scores in the patients in the cryotherapy (TC) group was significantly lower than the control group (p = 0.014). In other words, 67% of patients in the TC group did not report any pain, while 42.5% of T group patients had no pain. Patients’ satisfaction was compared in two groups by the Iowa questionnaire. This questionnaire had 11 questions; each question had six levels of answers so, the minimum and maximum scores were 11 and 66. The difference of these values between the TC (88/0 ± 48) and T (4/1 ± 49) groups was insignificant (p = 0.5). The surgeon described his satisfaction level as "excellent" in 20% and 5% of the patients of TC and T groups, respectively. At the same time, he mentioned a "bad" experience in 5% and 27.5% of the TC and T groups. The level of surgeon satisfaction in the TC group was significantly different from the T group (p = 0.01).

As shown in Table 1, there were no significant difference of sedation score values in cryotherapy and control groups. (p = 0.87). In other words, 47.5% of patients in the TC group and 45% of T group patients during surgery were complete relax.

**Table 1:** Distribution of the relative frequency of sedation level according to Ramsay score

Variable^a^	T group (n = 40)	TC group (n = 40)	P-value^ǂ^
Head and neck movement	8 (20)	7 (17/5)	
			0/879
Mild hand movements	7 (17/5)	9 (22/5)	
Patients groaning	7 (17/5)	5 (12/5)	
Complete relaxation	18 (45)	19 (47/5)	

T group = Patients received topical anesthesia

TC group = Patients received topical anesthesia –crayotherapy

^a^Data is presented as a number (percentage)

^**ǂ**^Chi-square test was used

Patients with sedation scores below four were given fentanyl at a dose of 1 μg/kg. The relative frequency of patients who received fentanyl was not significantly different between the TC [n = 21 (48.8%)] and T [n = 22 (51.2%)] groups (p = 1.000).

**Fig. 2:**
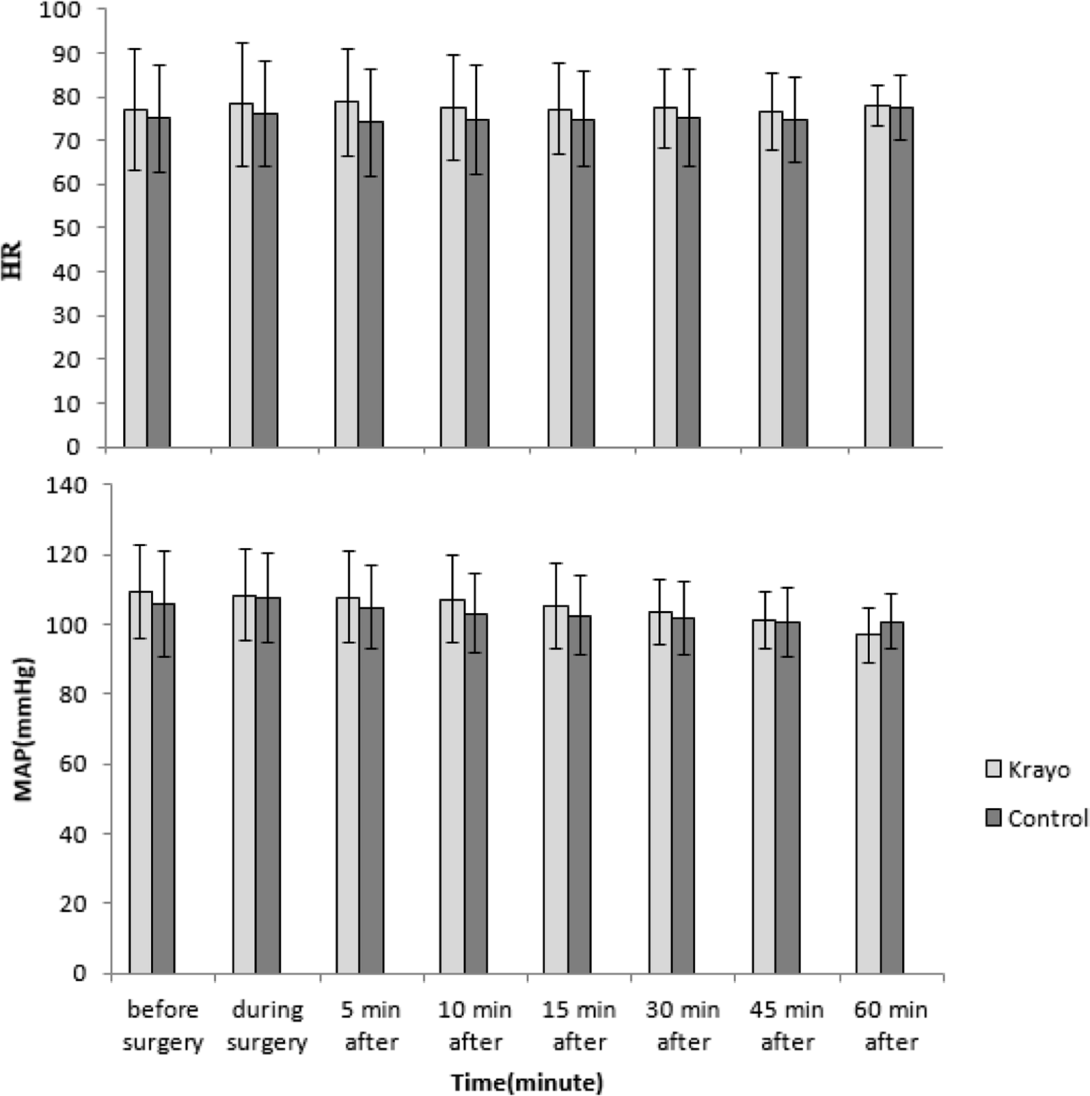
Hemodynamic variables

As shown in Fig. 2, the two groups were not significantly different in the hemodynamic evaluation, including heart rate (p = 0.347) and mean arterial pressure (p = 0.142).

No patient developed hypoxia, hypotension, bradycardia, excessive drowsiness (or sleepiness), and vomiting during the surgery. The postoperative side effects did not show a statistically significant difference between the two groups. Only one case of chills and one case of headache were recorded in the TC group. In the control group, we recorded three incidents of nausea and two cases of headache. [Data are in Additional file 1: Fig. S1, Additional file 2: Table S1, Additional file 3: Table S2, Additional file 4: Table S3, Additional file 5: Table S4].

### Discussion

The findings of our study revealed that cryotherapy is a complementary method with topical anesthesia and has a significant positive effect on pain intensity during surgery and surgeon satisfaction. This finding is partly consistent with a previous study by Gutierrez-Carmona et al. [4]. which declared that the pain level in **c**ryoanalgesia clear corneal phacoemulsification and topical anesthesia are almost equal. They suggested that cryoanalgesia is a suitable technique for anesthetic allergy cases [4].

While, in the Coelho et al. studies, which were performed on patients undergoing cataract surgery, no significant difference was observed in the amount of pain between the topical anesthesia versus topical combined to cryotherapy [8].

However, it seems that methodological differences in the studies can explain some of these contradictions. For example, the number of surgeons, surgery on one or both eyes, different groups of patients in terms of age and sex, different pain sensitivity and perception of patients and different tools to assess the amount of pain and also the type of medication prescribed to reduce patients' anxiety. Furthermore, the old age of patients and the lack of accurate answers to the questionnaire can also be another reason for the discrepancies in the results.

Nevertheless, it is known that cryotherapy having anti-inflammatory and analgesic effects [6]. Cryotherapy reduces the conduction velocity of the nerve fibers due to the asynchronous transmission in pain fibers, the release of endorphins, and the inhibition of spinal neurons, and an increase in the refractory period, which leads to a gradual lessening in the transmission of impulses in the sensitive nerves [11]. It has been suggested that the use of cold intraocular solution during the phacoemulsification, prevents the transmission of mechanical pain impulses continuously [11].

Another mechanism suggested for the analgesic effects of cryotherapy is cold-induced vasoconstriction, which can reduce blood flow to the target tissue and reduce the release of inflammatory mediators, and also, it promotes the accumulation of local anesthetic in the target tissue [6, 11]. Finally, the synergistic effect of cryotherapy along with topical anesthesia led to a greater sense of comfort in patients during surgery.

The other notable finding is that the level of sedation of patients in both groups was not statistically significant. That's probably, all patients in the ward were given 3 mg of melatonin one hour before entering the operating room.

In the same way, the patient satisfaction numbers (IOWA) were the simillar in both groups in our study.

Since the excessive sedation and decreased cognitive function leading to loss of patient cooperation with the surgeon, and also make the evaluation of patients' pain inaccurate. Thereby, to avoid bias, in the present study, one hour before entering the operating room, we used melatonin as an anxiolytic drug to increase patient satisfaction. In previous studies, the anxiolytic effect of melatonin without cognitive dysfunction was demonstrated as a pretreatment in various surgeries [3].

Another finding of this study was the greater satisfaction of the surgeon in the cryoanalgesia group. The lower pain scores of patients during surgery in the TC group seemed to be directly related to the surgeon's satisfaction.

Although, our study had some limitations; old age of patients and low level of their cooperation to answer the questions of the inevitable questionnaire. We recommend future studies in patients with bilateral cataracts.

### Conclusion

The findings of the present study revealed that cryotherapy as a complementary method with topical anesthesia could reduce patients' pain scores during surgery and enhance surgeon satisfaction.

## Limitations

Our study had some limitations; old age of patients and low level of their cooperation to answer the questions of the inevitable questionnaire. We recommend future studies in patients with bilateral cataracts.

## Supplementary Information

**Additional file 1: Fig. S1.** Consort flow diagram.

**Additional file 2: Table S1.** Demographic data.

**Additional file 3: Table S2.** Comparison of surgeon satisfaction in two groups.

**Additional file 4: Table S3.** The relative frequency of pain intensity in two groups.

**Additional file 5: Table S4.** Comparison of postoperative side effects in the two groups.

## Data Availability

The data that support the findings of this study are available on request from the corresponding author.

## Abbreviations

TC: Topical anesthesia with cryotherapy
T: Topical anesthesia alone
VAS: Visual analogue scale
ASA: American Society of Anesthesiologists
ISAS: Iowa satisfaction with anesthesia scale
SPO2: Pulse oximetry
NSAIDs: Non-steroidal anti-inflammatory drugs
LOCS: Lens opacity classification system
BSS: Balanced salt solution
