# Supplementary figures and images for: Effect of cryotherapy on pain scores and satisfaction levels of patients in cataract surgery under topical anesthesia: a prospective randomized double-blind trial
Source: BMC Res Notes. 2022 Jun 28;15:234. doi: 10.1186/s13104-022-06125-w (PMC9241292; doi:10.1186/s13104-022-06125-w)

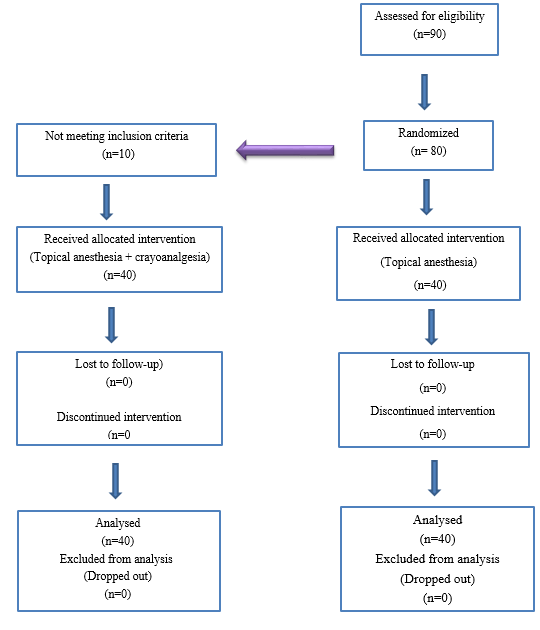


Additional file 1: Fig. S1. Consort flow diagram

Supplement: Supplementary file 1 — Additional file 1: Fig. S1. Consort flow diagram. [file 13104_2022_6125_MOESM1_ESM.docx]
