# Supplementary material for: Effect of cryotherapy on pain scores and satisfaction levels of patients in cataract surgery under topical anesthesia: a prospective randomized double-blind trial
Source: BMC Res Notes. 2022 Jun 28;15:234. doi: 10.1186/s13104-022-06125-w (PMC9241292; doi:10.1186/s13104-022-06125-w)
Supplement: Supplementary file 2 — Additional file 2: Table S1. Demographic data. [file 13104_2022_6125_MOESM2_ESM.docx]

Table S1. Demographic data

| P-value^ǂ^ | TC group  (n=40) | T group  (n=40) | Variable |
| --- | --- | --- | --- |
| 5/0 | 9/6 ± 1/66 | 2/6 ± 9/65 | Age |
| 13/0 | (5/42) 17 | (5/57) 23 | Gender** |
| 239/0 | 07/2 ± 8/10 | 87/1 ± 3/11 | Duration of surgery^*^ |

*Data are presented as mean ± SD. Independent samples t-test was used.

** Data are presented as number (%). Chi-square was used.

T group =Patients received topical anesthesia.

TC group= Patients received topical anesthesia –crayotherapy
