# Supplementary material for: Effect of cryotherapy on pain scores and satisfaction levels of patients in cataract surgery under topical anesthesia: a prospective randomized double-blind trial
Source: BMC Res Notes. 2022 Jun 28;15:234. doi: 10.1186/s13104-022-06125-w (PMC9241292; doi:10.1186/s13104-022-06125-w)
Supplement: Supplementary file 3 — Additional file 3: Table S2. Comparison of surgeon satisfaction in two groups. [file 13104_2022_6125_MOESM3_ESM.docx]

Table S2. Comparison of surgeon satisfaction in two groups

| P-value | TC group  (n=40) | T group  (n=40) | Variable |
| --- | --- | --- | --- |
| 01/0 | (20) 8 | (5) 2 | Excellent |
| - | (65) 26 | (5/57) 23 | Good |
| - | (10) 4 | (10) 4 | Moderate |
| - | (5) 2 | (5/27) 11 | Bad |

*Data presented as a number (percentage).

ǂ Chi-square test was used.

T group =Patients received topical anesthesia.

TC group= Patients received topical anesthesia –crayotherap
