# Supplementary material for: Effect of cryotherapy on pain scores and satisfaction levels of patients in cataract surgery under topical anesthesia: a prospective randomized double-blind trial
Source: BMC Res Notes. 2022 Jun 28;15:234. doi: 10.1186/s13104-022-06125-w (PMC9241292; doi:10.1186/s13104-022-06125-w)
Supplement: Supplementary file 4 — Additional file 4: Table S3. The relative frequency of pain intensity in two groups. [file 13104_2022_6125_MOESM4_ESM.docx]

Table S3. The relative frequency of pain intensity in two groups

| P-value^ǂ^ | TC group  (n=40) | T group  (n=40) | Variable |
| --- | --- | --- | --- |
| 014/0 | (5/67) 27 | (5/42) 17 | No pain |
|  | (30) 12 | (5/32) 13 | Mild pain |
|  | (0) 0 | (20) 8 | Moderate pain |
|  | (5/2) 1 | (5) 2 | Sever pain |

^ǂ^ Data are presented as number (%). Chi-square was used.

T group =Patients received topical anesthesia.

TC group= Patients received topical anesthesia –crayotherapy.
