# Supplementary material for: Effect of cryotherapy on pain scores and satisfaction levels of patients in cataract surgery under topical anesthesia: a prospective randomized double-blind trial
Source: BMC Res Notes. 2022 Jun 28;15:234. doi: 10.1186/s13104-022-06125-w (PMC9241292; doi:10.1186/s13104-022-06125-w)
Supplement: Supplementary file 5 — Additional file 5: Table S4. Comparison of postoperative side effects in the two groups. [file 13104_2022_6125_MOESM5_ESM.docx]

Table S4. Comparison of postoperative side effects in the two groups

| P-value | TC group (n=40) | T group (n=40) | P-value | Variable |
| --- | --- | --- | --- | --- |
| 2/0 | (5/2) 1 | (0) 0 | 2/0 | shivering |
| 9/0 | (5/2)1 | (5)2 | 9/0 | headache |
| 2/0 | 0 | (5/7) 3 | 2/0 | nausea |

*Data is displayed as a number (percentage).

ǂ Chi-square test was used.

T group =Patients received topical anesthesia.

TC group= Patients received topical anesthesia –crayotherapy
